# Multi-stakeholder sessions on major innovation topics in rare disease clinical trials

**DOI:** 10.1186/s13023-024-03482-6

**Published:** 2024-12-19

**Authors:** Daniel Bodden, Stefanie Schoenen, Stephanie Wied, Johan Verbeeck, Maya Dirani, Hiba Abou Daya, Nicole Heussen, Geert Molenberghs, Ralf-Dieter Hilgers, Rima Nabbout

**Affiliations:** 1 Institute of Medical Statistics, RWTH Aachen University Aachen, Pauwelsstrasse 19, 52064 Aachen, Germany; 2 I-BioStat, Data Science Institute, Hasselt University, Martelarenlaan 42, 3500 Hasselt, Belgium; 3 Medical School, Sigmund Freud Private University, Freudplatz 1, 1020 Vienna, Austria; 4 Institut des Maladies Gènètiques Imagine- Necker Enfants malades Hospital, 24 Boulevard du Montparnasse, 75015 Paris, France; 5 Necker Enfants malades Hospital, 149 Rue de Sèvre, 75015 Paris, France; 6 I-BioStat, KU Leuven, Kapucijnenvoer 35, 3000 Leuven, Belgium; 7 Thematic Institute of Genetics, Genomics & Bioinformatics, INSERM, 8 rue de la Croix Jarry, 75013 Paris, France

**Keywords:** Finite populations, Natural history, Randomization, Multiple endpoints, Educational system

## Abstract

**Background:**

The European Joint Programme on Rare Diseases aims to enhance the rare diseases research ecosystem by bringing together stakeholders such as research funders, institutions and patient organizations. Work Package 20 focuses on the validation, use and development of innovative methodologies for rare disease clinical trials. This paper reports on the outcomes of a retreat held in April 2023, where areas for innovation and educational needs in rare disease clinical trials were discussed in multi-stakeholder sessions.

**Methods:**

Multi-stakeholder sessions covered the topics: Future Educational System, Randomization in Rare Disease Clinical Trials, Endpoints in Rare Disease Clinical Trials and Using History Course Data. The sessions began with expert presentations to set the scene, followed by guided discussions facilitated by questions on a collaborative digital whiteboard. Participants wrote responses, which were then discussed live with the experts.

**Results:**

Training is needed for diverse stakeholders in rare disease clinical trials to enhance understanding and drive innovation. Challenges include a lack of standardized terminology for multiple endpoints, inadequate understanding of randomization in small sample studies and various obstacles in effectively using natural history data.

**Conclusion:**

Creating a comprehensive and sustainable educational program for rare diseases clinical trial methodology requires strategic collaboration and adherence to FAIR principles. The workshop highlighted the need for innovations for topics in areas such as handling missing data, optimizing the extraction of information from small samples, remote endpoint measurement and new randomized inference techniques. Additionally, integrating innovations into tailored training programs is crucial for advancing the field.

## Background

The European Joint Programme on Rare Diseases (EJP RD), which commenced in 2019 and concluded in August 2024, was dedicated to consolidate the Rare Diseases (RDs) research ecosystem with the aim of advancing patient outcomes through research progress and innovation. It brought together various stakeholders, such as research funders, ministries, research institutes, universities, European Reference Networks (ERNs), European Union (EU) research infrastructures, foundations, regulators and patient organizations [1]. The program extended its support to stakeholders in the RD community across four Pillars: 1. Research Funding; 2. Data, Resources and Tools; 3. Training and Empowerment; 4. Acceleration of Research Translation and Clinical Trials [1]. Within this structure, Work Package 20 (WP20) falls under Pillar 4 focusing on accelerating the validation, use and development of innovative methodologies tailored to RD clinical trials. As a result of WP20’s activities, research and clinical teams can find dedicated support in setting up clinical trials for their innovative projects. A specific form of assistance is tailored to help clinical investigators in preparing clinical trials aimed at developing novel treatments, re-purposing drugs or devices, and conducting diagnostic studies [2]. In the same vein, EJP RD launched two internal calls that resulted in the funding of three “Demonstration” and two Innovation” projects to improve outcomes of clinical studies in RDs. The three demonstration projects aimed to show the usability and capability of recently developed innovative statistical methodologies for clinical trials in RDs, which have not been demonstrated in existing data for specific RD clinical trials yet [3]. The two innovation projects were aimed at improvement and innovation of methodology for trials in limited-sized populations, for example, in relation to trial design, endpoints or the statistical analysis methods [4]. Furthermore, WP20 developed a structured educational program to disseminate advanced statistical trial methodology. It encompasses advanced webinars on methodology in RD clinical trials tailored to the needs of expert stakeholders; they were delivered as live webinars as well as intermediate courses tailored to diverse audiences and delivered in the format of pre-recorded lectures [5]. During a retreat held on April 13–14, 2023, we reviewed the current state of activities and discussed potential future developments. In collaboration with various stakeholders, we identified and analyzed key challenges that require attention moving forward. Based on these discussions, we formulated recommendations, addressing both educational and research needs, which we summarize in this paper.

This paper reports on the discussions and outcomes from the multi-stakeholder meeting, organized as follows. The Methods section outlines the structure of the sessions and their discussion formats. The Results section details the input gathered, combined with discussions from each session, organized into subsections. Lastly, the Summary and Conclusion section highlights key recommendations and outlines future plans.

## Methods

The second day of the retreat was dedicated to multi-stakeholder sessions on key topics related to innovation in RD clinical trials. The meeting participants represented a broad spectrum of stakeholders involved in rare disease research and clinical trials. Invitations were sent to all ERNs via their coordinators and to representatives from industry, patient organizations and members of the EJP RD. In total, 30 participants attended in person, including six representatives from ERNs, two from European Patient Advocacy Groups (ePAGs), six from the pharmaceutical industry, and ten from academic institutions with both clinical and methodological expertise. Additionally, six participants were from broader research infrastructures like the European Clinical Research Infrastructure Network (ECRIN). The participants originated from several European countries, including Austria, Belgium, Germany, France, the Netherlands, Italy, and the UK, thus incorporating the perspective of multiple healthcare systems and experiences in rare disease management. The diversity of stakeholders is crucial to the subsequent discussions, as it reflects the collaborative and multidisciplinary nature required to advance innovation in rare disease clinical trials.

The sessions were designed to encourage comprehensive discussion among stakeholders. To ensure that each stakeholder could engage fully in each topic, the sessions were held consecutively. The organization of these sessions was as follows:

1. Future Educational System,
2. Endpoints in Rare Disease Clinical Trials,
3. Randomization in Rare Disease Clinical Trials,
4. Using History Course Data.

Each session began with a “setting the scene” presentation by experts in the particular area under consideration. These presentations aimed to provide contextual background and establish a foundation for subsequent discussions. The main messages from these expert talks are elaborated upon in the corresponding subsections of this paper. To stimulate and structure the discussions, the first three sessions included guiding questions formulated by the experts. These questions were strategically chosen to provoke thought and stimulate meaningful dialogue. The questions were displayed on a collaborative digital whiteboard, accessible to all participants. Participants were given three minutes to respond to each guiding question by writing their thoughts on the digital whiteboard. This interactive method ensured that all voices were heard and that a wide range of ideas could be captured quickly. Following the initial note-taking phase, the experts facilitated lively discussions based on the notes collected. These discussions allowed participants to elaborate on their points, challenge each other’s views and collectively explore potential solutions. The notes from the collaborative digital whiteboard are included in the appendix.

## Results

### Future educational system

#### Setting the scene

**Fig. 1:**
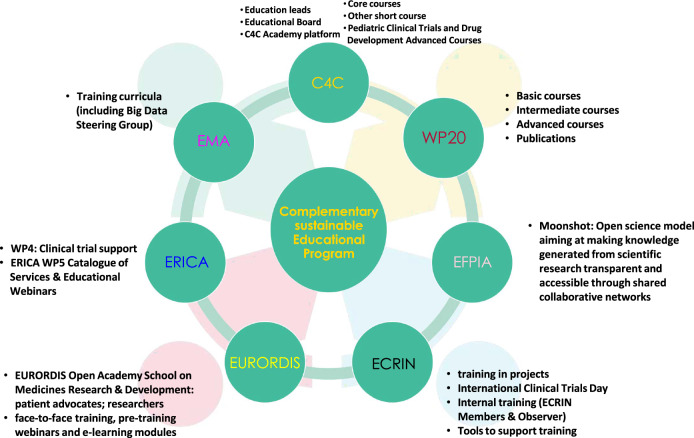
An overview of the educational programs for rare disease clinical trials methodologies. Key components include various institutions active participation such as the European Medicines Agency (EMA), connect 4 children (C4C), Work Package 20 (WP20) of the European Joint Programme on Rare Diseases, European Federation of Pharmaceutical Industries and Associations (EFPIA), European Clinical Research Infrastructure Network (ECRIN), European Organisation for RD (EURORDIS), and European Rare Disease Research Coordination and Support Action (ERICA). Educational programs in RD Clinical Trial methodologies. This figure provides an overview of the various components within the Complementary Sustainable Educational Program under the WP20 initiative. It highlights key educational offerings such as training curricula, the C4C Academy platform, and various levels of courses (basic, intermediate, advanced). The figure also outlines the contributions of different entities including connect 4 children (C4C), European Federation of Pharmaceutical Industries and Associations (EFPIA), European Clinical Research Infrastructure Network (ECRIN), European Organisation for RD (EURORDIS), and European Rare Disease Research Coordination and Support Action (ERICA). Additionally, it details specific programs like the Moonshot initiative, which aims to enhance the transparency and accessibility of scientific knowledge through collaborative networks. Other features include face-to-face training, pre-training webinars, e-learning modules, and a range of specialized courses tailored to support clinical trial training

The landscape of clinical trials in the field of RDs is rapidly evolving, necessitating a robust educational framework. Various organizations provide different educational tools for RD clinical research (see Fig. 1), which are summarized in the following.

WP20 offers a diverse range of educational resources tailored to meet the needs of learners at various levels of expertise. This includes: basic courses covering fundamental concepts; intermediate courses aiming to deepen learner’s knowledge on more nuanced topics; and advanced courses for experienced professionals on the topics Randomization, Composite Endpoints, Surrogate Endpoints and Master Protocols [5]. Furthermore, WP20 published several articles and educational materials that serve as valuable resources for learners seeking in-depth knowledge on specific topics and offering insights from leading experts [6].

The European Medicines Agency (EMA) enhances medical research and treatment protocols through guideline development and training initiatives, targeting regulatory processes and expert education. Guidelines cover various areas like Clinical Pharmacology, Real-World Data, Clinical Trial Modernization, Artificial Intelligence, Data Science, and Pharmacogenomics for precision medicine. The EMA Methodology Working Party offers a comprehensive working plan, openly accessible, detailing methodologies and presentation materials. Additionally, the Data Steering Group focuses on developing a big data training curriculum to strengthen regulatory expertise.

The European Rare Disease Research Coordination and Support Action Consortium (ERICA) Work Package 4 on Clinical Trial Support is actively involved in producing webinars and YouTube videos to share knowledge and best practices in various aspects of RD clinical trials. Collaborating with the EMA and the International RD Research Consortium (IRDiRC), ERICA ensures alignment with regulatory standards and international research initiatives. ERICA’s Work Package 5 is dedicated to creating a comprehensive catalog of services and educational webinars for the RD community. It also offers guidance on using essential tools developed within EU-funded initiatives, such as the Catalogue of Services and the Innovation Management Tool, to improve research efficiency. Additionally, ERICA conducted a survey to identify educational and research needs within ERNs. Furthermore, ERICA contributes to disseminating clinical practice guidelines.

The European Clinical Research Infrastructure Network (ECRIN) training programs focus on multinational aspects, developed through projects such as Vaccelerate, Personalised Medicine Trials (PERMIT), ERA4Health, and The Curriculum Development of Human Clinical Trials for the Next Generation Biomedical Students (CONSCIOUS). Vaccelerate is a clinical research network dedicated to advancing the conduct and innovation of vaccine trials [7]. PERMIT is an EU-funded project aimed at establishing methodological standards for personalized medicine research. ERA4Health supports health research across Europe and CONSCIOUS is an Erasmus+ project addressing skill gaps in European-level clinical trial professionals. ECRIN also hosts events like the International Clinical Trials Day and ECRIN Clinical Trials Day, alongside scientific meetings, and offers specialized tools such as the RD Clinical Trials Toolbox and Adaptive Platform Trials Toolbox. The RD Clinical Trials Toolbox is designed to support investigators and sponsors conducting clinical trials in the field of RDs by providing guidance on the unique regulatory, ethical, and operational challenges these studies face, and the Adaptive Platform Trials Toolbox serves as a comprehensive resource for researchers implementing adaptive platform trials. ECRIN disseminates these resources through its website, press releases, social media, and various scientific platforms. They ensure that project-specific training materials reach stakeholders effectively through collaboration, partnering with national entities and clinical working groups for wider dissemination across European regions.

The European Federation of Pharmaceutical Industries and Associations (EFPIA) plays a vital role in disseminating project outcomes through educational initiatives and training programs for diverse stakeholders, including patient organizations. EFPIA engages with Health Technology Assessment bodies and addresses communication strategies early in projects, resulting in collaborative efforts such as the development of guide books, to showcase best practices and successful collaborations between healthcare stakeholders, patient organizations and industry partners across Europe [8]. EFPIA also collaborates on guidance document development, scaling up innovation manuals and initiatives such as the Moonshot project, which focuses on accelerating research through public-private partnerships on the world’s rarest and severe conditions that currently do not have therapeutic options.

The Connect4Children (C4C) initiative offers a comprehensive education program in pediatric clinical research, overseen by Education Leads and guided by the Educational Board. The C4C Academy Platform provides a virtual learning environment with course materials, forums and assignments. Core courses cover fundamental pediatric clinical trial topics, supplemented by short courses on specialized areas like developmental pharmacology. An advanced course delves into advanced pediatric drug development and regulatory issues. Accredited by Continued Professional Development (CPD), discussions are ongoing regarding the program’s sustainability model.

The European Organisation for Rare Diseases (EURORDIS) leads the EURORDIS Open Academy School on Medicines Research & Development, empowering patient advocates and researchers to actively contribute to medical research, particularly in RDs. By educating patients about clinical trials, EURORDIS enables informed decision-making, encouraging active participation and collaboration in trial design, ultimately enhancing care quality for RD communities. As a prominent advocate and resource hub, EURORDIS provides educational materials, workshops, webinars and campaigns, fostering patient engagement and collaboration among patients, researchers and healthcare professionals. Additionally, EURORDIS offers online courses covering various aspects of clinical trials and statistical methodologies.

Following this overview on selected key educational aspects the discussion was structured around four guiding questions:

1. Given the list of audiences for whom the educational programs are developed, do you think that we are missing (or under-serving) some groups?
2. What are in your opinion the uncovered needs for education in clinical trials?
3. What are the pros and cons of different formats? What is the best format and for which audience?
4. Sustainability/Accreditation: How to make these programs sustainable (Continuing Medical Education, University, Scientific organisations, ...)?

#### Outcomes of the discussion

The discussion revealed that certain groups are underrepresented in clinical trials education, such as personnel from the pharmaceutical industry, specialized nurses involved in advanced care programs having direct interactions with the patients and families and different professionals who provide indirect support to clinical trial units. It was emphasized that basic courses should target professionals who have had limited exposure to clinical research, while advanced courses should be tailored to those with more specialized knowledge or extensive experience in the field. Tailored training is required for all researchers and physicians working on RDs. Additionally, addressing the educational needs of evaluators, researchers from less-represented countries, engaging with funders and regulatory bodies are essential for a comprehensive approach to clinical trials education. Attendees identified important gaps in clinical trials education, emphasizing the need for a more comprehensive framework. They stressed the importance of tailored programs focusing on competence-based and adaptive learning, alongside addressing patients’ educational needs to understand trial complexities. Key elements include Good Clinical Practice (GCP) training, disease-specific education, ethical considerations, and protocol development. Tailoring programs for different researcher populations while integrating modern technologies like artificial intelligence into the training is crucial. Bridging gaps in advanced methodology, operational aspects, regulatory classifications, and RD training is also highlighted. Additionally, ensuring program visibility, accessibility, budgeting, regulatory understanding, and tracking Continuing Medical Education (CME) points are vital for a holistic approach to clinical trials education. Analyzing various educational formats is crucial for catering to diverse audiences effectively. Short YouTube videos with info graphics involve the general public, while virtual lectures offer flexibility. The inverted classroom format promotes interactive learning and expert contact is vital. Alternative platforms like TikTok and Instagram are suggested, particularly for younger professionals, alongside preferences for short webinars and online programs. Interactive online courses and blended learning cater to different preferences, emphasizing adaptability and balancing online and in-person formats. Harmonizing accredited programs, flexible time slots and gamified experiences to increase engagement and motivation of the learner enhance the educational strategy’s versatility and effectiveness. Ensuring the sustainability and accreditation of future educational programs in clinical trials requires a multifaceted strategy. Agreements with universities anchor programs, while CME points enhance participant professional development. Innovative models like executive master programs and joint online seminars cater to diverse preferences, with a focus on regular updates to incorporate new research findings. Integration into specialization programs at national and European levels aligns with harmonization efforts. A robust business model, including sustainable funding sources, is crucial, alongside connections with entities like ERNs and research infrastructures. Diversification in sustainability approaches, openness to industry participation and leveraging social media and training platforms enhance accessibility. Tailoring programs to audience needs and fostering collaborations ensure a holistic and sustainable approach to clinical trials education.

### Multiple endpoints in rare disease clinical trials

#### Setting the scene

Proving effectiveness of novel treatments in RDs is challenging. Besides the issue of limited populations, RDs show multiple phenotypes, have heterogeneous clinical presentations, often age-related, and the natural history is typically still poorly understood. This complicates the definition of one or more endpoints for the evaluation of a treatment. “Developing endpoints that meet the scientific and regulatory best practices has become a central challenge in the development of novel methodologies in RDs” [9]. The regulatory side also recognizes that the development of new endpoint methodologies is key to the evaluation of innovative treatments in the field of RDs. Therefore, the U.S. Food and Drug Administration (FDA) has established in October 2022 a Rare Disease Endpoint Advancement Pilot (RDEA) Program to support the development of novel endpoint concepts for RD [10]. RD trials often rely on multiple endpoints to capture the full complexity of the condition and provide a more comprehensive assessment of treatment effects [11]. Both the EMA and the FDA have issued guidelines on the use of multiple endpoints, offering several strategies for incorporating them into clinical trials [12, 13]. Multiple primary endpoints involve several primary endpoints that are analyzed independently. A significant treatment effect on any of these endpoints may be taken as evidence of efficacy. Multiple primary endpoints are considered co-primary when each endpoint must demonstrate a treatment effect to establish efficacy, and in such cases, multiplicity is not a concern [14]. Composite endpoints combine multiple outcomes into a single variable, and treatment efficacy is assessed based solely on this composite. This approach is commonly used in time-to-event analyses, where the composite endpoint is defined as the first occurrence or realization of one of the pre-specified events in a subject [14]. Multi-component endpoints are a within-subject combination of two or more components into a single score or rating, whose attributes can be either weighted or unweighted [14]. Challenges of different endpoint types and their evaluation methods in the field of RDs are discussed in [15].

To define future areas of research, it is necessary to discuss the problems researchers encounter when using multiple endpoints. Therefore, the following questions guided the discussion:

1. What issues challenge you in using multiple endpoints in your research?
2. What innovations regarding multiple endpoints are necessary for RD clinical trials?
3. Which types of multiple endpoints are most helpful in the context of your RD clinical trials?

#### Outcomes of discussion

One crucial challenge for the use of multiple endpoints in RD clinical trials is the lack of standardized terminology, not just within published research and guidelines but also among the regulatory agencies. Another concern is the adoption of innovative statistical methods and their associated endpoints. The harmonization of terminology, the establishment of a standardized endpoint qualification process and the provision of guidance and education are essential. The absence of such standardization and education has left some workshop participants unable to identify the most suitable type of endpoint for their RD clinical trials. A major hurdle in RD clinical trials is the absence of validated endpoints. In contrast to common diseases where the natural history and clinically relevant endpoints are well-documented and accepted by all stakeholders, RDs, due to their infrequent occurrence and limited data, have largely unknown natural histories as well as optimal endpoints for assessing intervention effects. On the one hand, selecting outcomes for multiple endpoints should be based on a coherent understanding of the disease’s underlying biological processes and the mechanism of action of the intervention being evaluated. Ideally, these multiple outcomes should exhibit minimal correlation, which should be investigated in natural history studies of the disease. On the other hand, in the realm of RDs, the chosen endpoints and statistical methods should aim to maximize power with the smallest possible number of subjects, while maintaining interpretability of the global effect and the impact on individual outcomes. This task is further complicated by disease heterogeneity and variations in how endpoints are measured, potentially reducing the power. Identifying relevant subgroups with tailored endpoint adjustments can enhance homogeneity, power and reduce the required number of subjects. Regardless of the uncertainty surrounding which endpoints are relevant for capturing clinical benefits, there is growing interest in patient-centered outcome measures (PCOM) and patient-reported outcomes (PRO). This poses statistical challenges in how to evaluate both quantitative clinical outcomes and qualitative outcomes, like quality-of-life measurements, together. While various methodologies have been proposed, further innovation is needed to optimally combine different types of outcomes and determine which patient-centered outcome measures genuinely reflect clinical benefit rather than serving as proxies. To enhance the identification of endpoints for RD clinical trials, it is imperative to investigate the natural history of the disease and understand the potential association between outcomes, followed by collaboration with regulatory authorities for endpoint qualification. Both agencies have processes to assist in qualifying novel endpoints [9]. This qualification hinges on the alignment of endpoints with biological processes and their consistency of use throughout the entire drug development program. Once relevant outcomes are selected, additional research and education, including practical examples, are necessary to provide guidance on how to jointly analyze multiple endpoints. This analysis can be achieved through separate testing with multiplicity correction, weighting, ranking, joint modeling, item response theory, goal attainment scales, or other methodology. Education should target all stakeholders, including clinicians, statisticians, regulatory authorities and patients.

### Randomization in rare disease clinical trials

#### Setting the scene

**Fig. 2:**
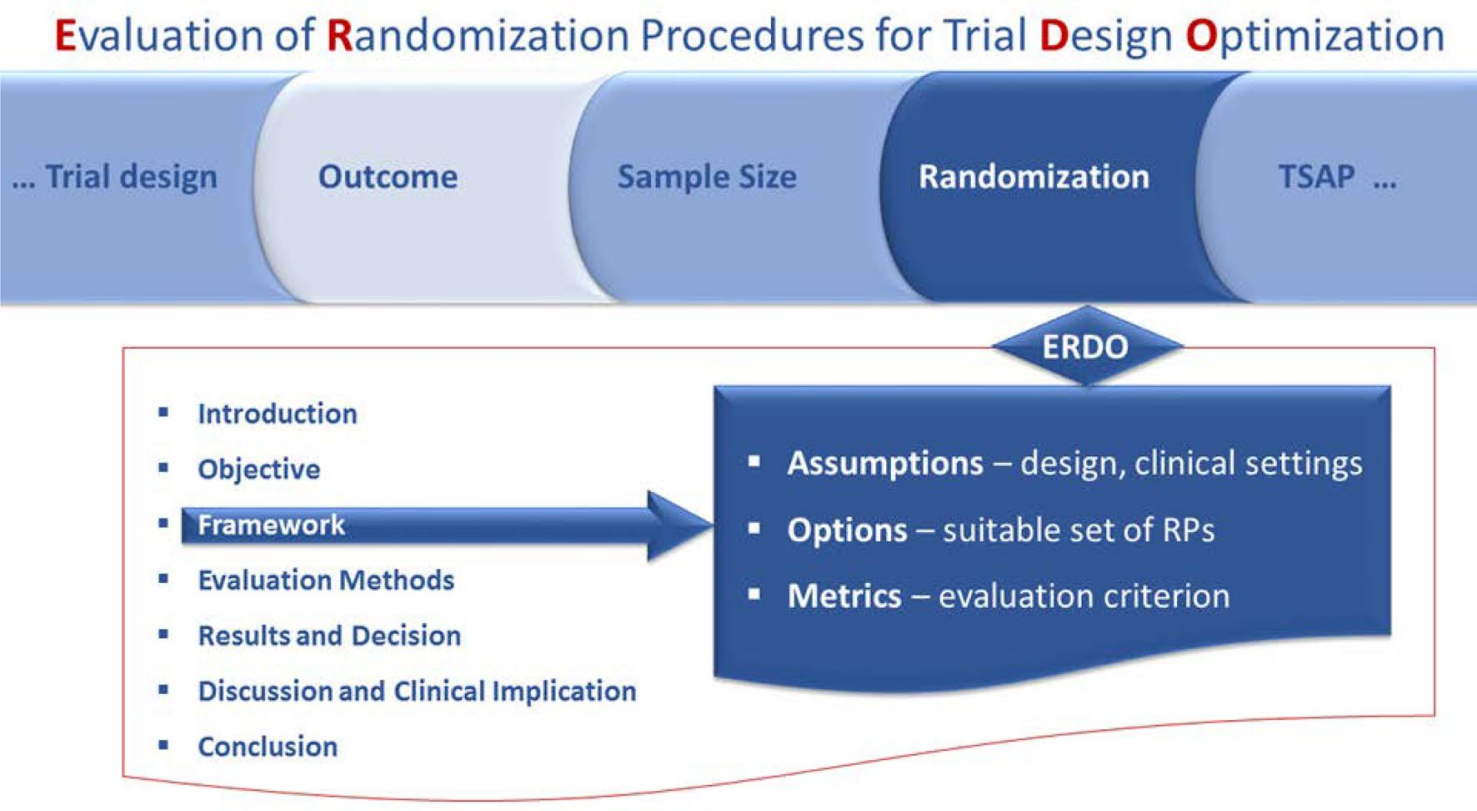
Evaluation of randomization procedures for design optimization (ERDO) framework. This figure describes the ERDO framework, which is used to identify the best randomization procedure. The framework incorporates key aspects of the trial, including disease types, outcomes, and other relevant factors to find the best randomization procedure in the planning phase of the clinical trial

In addition to blinding, randomization is the key approach to avoid bias in comparative confirmatory and exploratory clinical trials. It is worth noting that randomization can be implemented without blinding. As randomization is supposed to mitigate bias, it increases validity of the study findings. This technique is not limited by the size of the study nor the size of the target population under study. However, it appears that the potential to mitigate bias depends on the setting of the clinical trial, in particular the sample size as well as the type of the randomization algorithm, a.k.a. procedure. Randomization is the process of random allocation of treatments to patients in trials. Consequently, random sampling is not a feature of the random allocation process. On the other hand, the usual inference relies on the assumption of random sampling only and some inference procedures need particular features of the random allocation process, e.g., balanced sample sizes. The mix up of random sampling and random allocation results in confusion and is a source of criticism e.g., either for “external validity” where random sampling might be conflicted, or predictability, where random allocation is subverted. The problem of bias is recognized by the regulators, who state that it operates despite the best intentions of sponsors and investigators and may lead to flawed conclusions [16, 17]. Accordingly, the effect of bias on the trial results should be gauged by sensitivity analyses. Over the last decades, considerable efforts have been made to enable such sensitivity analyses, particularly in the context of small population clinical trials, and to provide a software solution for performing them [18, 19]. The approach can be used to identify the most efficient randomization procedure in the planning phase of the trial, realizing that there is no “one size fits all” procedure. The selection is based on a comprehensive simulation study, called ERDO [18] prior to study commencement to identify the randomization procedure for the specific study design similar to the clinical scenario evaluation tool [20], see Fig. 2. However, random allocation can also be used to evaluate the level of evidence in the data. This approach, i.e.,randomization-based assessment of the level of evidence, is based on trial simulation at the design stage in situations where the trial population is finite and small. This setting presents challenges, as independence of sampling is not plausible and sample size could not be extended in a reasonable time. Guidance on what is meant by “small” needs to be developed in the future. Conclusions should be drawn in accordance with the randomization procedure used. In addition, it should be clear what level of evidence is required for a treatment to be considered successful in a particular trial.

Past and present challenges as well as future development areas were the focus of the discussion, which was guided by the following three questions:

1. What are practical problems with implementation of randomization in RD clinical trials?
2. Is the current state of knowledge about implementation of randomization sufficient in the RD community?
3. What are needs for innovation with randomization?

#### Outcomes of discussion

During the discussion, several misconceptions regarding randomization in clinical trials that require educational attention were addressed. One prominent misconception was the belief that being part of the placebo or active control group is a “waste”. To clarify, this assumption is based on the belief that the drug being tested is effective. However, if the drug is found to be ineffective, the opposite would be true. The importance of patients understanding the value they contribute to the community by participating in a trial, particularly when assigned to the placebo group, was emphasized. To counteract this misconception, it was recommended to highlight the benefit to the patient population as a whole.

The challenge of conducting sample size calculations when there is insufficient data available was also discussed. It was explained that this challenge pertains to confirmatory clinical trials. However, in exploratory trials, sample size considerations are based on different considerations. The lack of awareness regarding this distinction within the community was highlighted. Furthermore, the discussion delved into the complexity of trial designs, particularly in comparison to textbook examples, with the pharmaceutical industry being more proficient in implementing multicenter studies.

It was emphasized that raising awareness about the possibility of allocation bias [21] is necessary, because many people perceive randomization as an infallible gold standard without room for error or bias. It was suggested that quantifying bias, recognizing its varying prominence depending on the situation and providing education on the limitations of randomization are crucial in addressing this misconception.

The issue of randomization preventing the sharing of placebo group information was raised and the Bayesian approach was acknowledged as less understood in this context. Additionally, it was noted that clinicians have limited knowledge of Bayesian approaches.

The topic of innovation in randomization was also brought up, questioning whether new approaches should be explored in the era of open registries and observational real-world data, particularly in the context of RDs. It was emphasized that the line between randomized experiments and observational studies is becoming blurred and a combination of both approaches could be beneficial. While some argue that randomization is no longer necessary due to better causal inference, it was concluded that properly designed randomized experiments remain essential. However, incorporating observational data in data-poor areas would be valuable. In relation to this, it was noted that the EMA recently published a guideline on registry-based studies [22].

The discussion also raised ethical concerns regarding certain trial scenarios, such as the burden of intervention on young children. It was proposed that subjecting children to interventions for extended periods might be unethical. Factors such as the type of exposure and the child’s age were suggested as important considerations in such cases.

### Using natural history course data

#### Setting the scene

Two stimulating presentations were delivered by experts from both the methodological and patient representative sectors, who contributed to the WP20 demonstration and innovation projects. These experts provided a summary of their findings, drawing from their work on the IMPROVE PSP and EVIDENCE-RND projects [3, 4]. Their presentations offered an overview of recent achievements and key outcomes, setting a foundation for the discussion.

#### Outcomes of discussion

The focus of the discussion was on the potential beneficial interaction between the clinical trial framework and the natural history framework. It was emphasized that we, as researchers, owe society – especially in the area of RDs – to extract as much information as possible from individual patient data, especially when a control arm may not be feasible due to large estimated effect sizes or lack of equipoise, necessitating reliance on single-arm studies and historical controls. However, these endeavors face notable challenges. One challenge is given by the privacy regulations like the General Data Protection Regulation (GDPR), which are more impactful in areas such as RDs. Therefore, the availability of individual patient data instead of aggregated datasets should be an academic as well as commercial commitment. The marketing authorization for RD medicine is often difficult to obtain. In such cases, it is important to collect historical and clinical data all around Europe, but the datasets are very different and difficult to use for qualitative purposes. There was a call for early collaboration among researchers, regulators, health technology assessment bodies, clinicians and patients to establish appropriate outcome measures for studying natural disease progression through pre-competitive research. On the other end of the spectrum, the necessity of long-term follow up was raised and the question of logistical and financial challenges for those were discussed. It was noted that the pricing for long-term follow up might be difficult to be entered in the pipeline of the development and pricing of the drug and that the regulator has to play a vital role in that area. Solutions such as virtual institutes or patient reported outcomes via “smart cards” were mentioned to facilitate long-term data collection.

One question that was raised was: What really is natural history data? It is proposed that natural history data should not be viewed in isolation, but in connection with statistical, clinical, biological data types and various modeling approaches. Models like Markov chain models, Item Response Theory models and linear mixed models must be considered in this regard. Also, an irregularly spaced measurement schedule is a very defining characteristic of natural history data. The fact that people may start getting measured late, when the disease has already manifested and leave early, for example, when the disease is getting worse, do not get measured at all times and that there is missing data must be addressed. It was emphasized that bias does not necessarily invalidate research findings, instead it requires careful consideration. It may be that certain aspects cannot be studied anymore, but on the other hand, the natural heterogeneity can be a very interesting source to study in the first place. How frequently people get measured may already carry information by itself.

## Summary and conclusion

**Table 1:** Recommendations for future educational systems and innovation of rare disease clinical trials

Session	Key recommendations
Future educational system	1 Development of specific training including GCP, disease-specific education, ethical considerations, and protocol development tailored for different populations
	2 Develop methodological trainings for topics such as randomization, multiple endpoints, and natural history data analysis
	3 Developing innovative educational formats tailored to meet the diverse needs of different audiences
	4 Develop a robust business model guaranteeing sustainable accessibility of the training programs
Randomization in rare disease clinical trials	5 Raise awareness among the patient population about the importance of randomization in clinical trials and its benefits to reach valid evidence
	6 Increase awareness of possible biases and methods to quantify bias on study results
	7 Develop hybrid design technologies combining randomized clinical trials with observational data
Endpoints in rare disease clinical trials	8 Establish standardized terminology and a regulatory framework on how to handle multiple outcome data
	9 Develop and validate disease-specific (multiple) endpoints considering various measuring scales in cooperation with regulatory authorities
	10 Develop appropriate analysis procedures and guidance on their use in cooperation with regulatory authorities
Using history course data	11 Extract as much information as possible from individual patient data to complement clinical trials
	12 Enhance early collaboration to establish the same outcome measures and standardized FAIR clinical databases
	13 Learn from natural heterogeneity of data collection about disease characteristics

The following table 1 summarizes the recommendations derived from the discussions within each session. These aspects will be addressed in two major EU-funded projects, namely ERDERA (grant agreement No. 101156595) and RealiseD (grant agreement No. 101165912).

The retreat meeting could not cover all aspects of innovation in RD clinical trials. For instance, innovative trial designs, such as platform trials, were not addressed. Additionally, while the discussion on endpoints focused primarily on the challenges associated with multiple endpoints, the need to develop more sensitive endpoints for RDs, as well as to explore innovative approaches, including digital markers and AI-based assessments for endpoints, would benefit from discussions in the future. Regulatory agencies needs for endpoints in rare and ultra rare diseases will be considered in the upcoming RealiseD project and thus our discussion could not present any solutions here. The use of multiple endpoints in RD clinical trials challenges researchers due to the lack of standardized terminology and the absence of recommendations on how to define and evaluate meaningful patient-centered endpoints in small populations. There is still a lack of understanding in the role of randomization in RD clinical trials, particularly in trials with limited sample sizes. Randomization also needs to be understood in hybrid studies, combining randomized experiments and observational studies.

Overcoming regulatory, methodological and privacy regulatory barriers to fully leverage natural history data necessitates a collaborative and integrated research approach for the RD community. Reflection on natural history studies would also benefit from consideration of the many RD history studies that exist in Europe and beyond. An example can be found in [23].

Creating the best comprehensive and global educational program in RD clinical trial methodology necessitates strategic collaboration among various organizations offering educational tools. By taking advantage from each entity’s strengths and fostering collaboration, a cohesive educational ecosystem can address the diverse needs of stakeholders in RD research and clinical trials. To achieve this, a multi-faceted approach to collaboration can be implemented. Initially, organizations must identify complementary resources and assess their compatibility. Subsequently, a framework for integration is established, mapping out curricula and developing pathways for learners across different organizations. Collaboration manifests itself in joint training initiatives, co-development of materials and shared resources. Additionally, knowledge sharing and exchange are promoted through cross-organizational training programs and collaborative platforms. Lastly, establishing governance and oversight mechanisms ensures the success and sustainability of collaborative efforts by coordinating activities and monitoring progress. The future educational plan must be a FAIR one, aiming to ensure that the educational system for RD clinical trials is Findable, Accessible, Interoperable and Reusable. This entails implementing principles of open science and digital innovation. Efforts include making educational resources discoverable through centralized platforms with user-friendly interfaces and robust search functionalities. Accessibility is enhanced by removing barriers such as language and geographic restrictions, offering multilingual support and designing responsive interfaces. Interoperability is crucial for seamless resource integration, achievable through common standards for content formatting and metadata description. Promoting reuse involves designing materials in modular formats and adopting open-access policies. Adhering to these principles can maximize the educational system’s impact, empowering stakeholders and driving advancements in research and clinical practice in RD.

## Supplementary Information

Additional file 1.

## Data Availability

The authors declare that the data supporting the findings of this study are available within the papers supplementary files.

## Abbreviations

EJP RD: European joint programme on rare diseases
RD: Rare diseases
ERN: European reference networks
EU: European union
WP20: Work package 20
EMA: European medicines agency
ERICA: European rare disease research coordination and support action consortium
IRDiRC: International rare diseases research consortium
ECRIN: European clinical research infrastructure network
EFPIA: European federation of pharmaceutical industries and associations
ACT EU: Accelerating clinical trials in the European union
C4C: Connect4Children
CPD: Continuing professional development
EURORDIS: European organisation for rare diseases
CME: Continuing medical education
FDA: Food and drug administration
RDEA: Rare disease endpoint advancement
PCOM: Patient-centered outcome measures
PRO: Patient-reported outcomes
ICHE9: International council for harmonisation E9
ERDO: Efficient randomization design optimization
GDPR: General data protection regulation
