# Supplementary material for: Multi-stakeholder sessions on major innovation topics in rare disease clinical trials
Source: Orphanet J Rare Dis. 2024 Dec 19;19:467. doi: 10.1186/s13023-024-03482-6 (PMC11657468; doi:10.1186/s13023-024-03482-6)
Supplement: Supplementary file 1 — Additional file 1. [file 13023_2024_3482_MOESM1_ESM.pdf]

Session 1 – Future Educational Systems

Audience: After the list of the audience for whom the educational programs are addressed, do you think that we are missing (or under addressing some groups)?

- Are industry personnel allowed to access?
- Health professionals (nurses etc) in general to support clear communication to patients
- Professionals from the industry, not directly working within CT units, but still adding value through the cross-functional collaborations. Basically the ones overtrained for the basic courses, but undertrained for the advanced ones.
- Pharmacologist
- Executives responsible for facilitating good infrastructure
- HTA
- GCP inspectors (in the broad sense)
- Financial controllers, how to accurately assess budgets
- Train nurses
- Train students
- Train clinicians
- Train the evaluator
- Needs of researchers from so called less represented countries
- Training of clinicians
- Funders
- Regulatory

Covered needs: What are in your opinion the uncovered needs for education in CTs?

- More tailored programmes – competence based – adaptive learning
- For patients: more insight into the complexity of CT
- GCP Training
- Diseases specific training on what is to study when
- Personal training – in the direction of consultation
- Ethics and consents issues of studies and trials
- 1. Functional: How to work with protocols
- 2. Design: How to contribute to protocol development
- 3. Expert: details of specific aspects (selection of randomization method)
- Need to cover general researchers population in diseases area congress (EULAR, …)
- Specific needs for academic sponsors
- Share successes and also what does not work
- Disseminate success stories
- Use modern technologies/AI
- Ethics and consent issues of studies and trials
- Training clinicians, including those not directly involved in CT, so that they can identify subjects that could benefit from CT
- Close connection between advanced (adaptive) methodology and operations
- Design of clinical trials and studies and their regulatory classification
- Better training of authority bodies on rare diseases and small populations, requirements, challenges

- visibility & accessibility to existing programs. as well as languagebarries for patients and site personnel
- building budgets as a PI of an academic trial
- learn about regulatory differences between countries
- visibility and tracibility and CME points

Format: What are the Pros and Cons of different formats? What is the best format and for which audience?

- General public: short YouTube videos with infographics
- A level of pragmatism needed: record and share virtually as lectures, can always be followed by more.
- Inverted classroom format
- expert contact is important
- meet the expert format either online or in person
- Usually, the courses are lengthy and boring... This could become particularly challenging when training younger professionals (for example (Gen Z) raised on social media. You could think of alternative channels e.g. TikTok, Instagram - check the ChemicalKim example. She is a Prof from the US, brilliantly explaining chemistry!
- prefer many short items that a long one
- Shorts webinars and online programs are preferred in my opinion. It is difficult to attend onsite courses
- blended learning (very good for heterogeneous audience)
- with use cases/share learnings
- peope only go in a MOOC if they are already convinced about the topic
- we must strat with something VERY SIMPLE and attention catching
- interactive online course with different cases for PhD students
- webinars - interactive: good for understanding, bad for revision
- peope only go in a MOOC if they are already convinced about the topic
- the aims of the training need to be adapted to the audience (learner). There is of course benefits to having training continuously available (online) but local training also brings with it other benefits - a different way to get to know the learning community. mix methods can be good or variations for differenty type of learner
- pre- recorded designed online course's you can follow on your own tempo/time
- blended learning concept has been developed for EBStatMax as "use case"
- how do we know different courses aren't overlapping one another?
- online and self- learning courses for students
- Depends on the educational goals of the course
- We need one result focused session to highlight importnace of learning about it
- It strongly depends on objective (info or real hands on learning): prepare flexible mixes (blended learning)
- In addition to format: consiedr multi teacher options (difernet perspectives)
- flexible in time slots - choose when you have the time and build up your credits
- adaptive formats, like a game with different levels
- Basic formats to be online, more in depth courses in person.
- different formats are fine , but there should be a Europe wide harmonised accrrediited programme overview

Sustainability/Accreditation: How to make these programs sustainable (CME? University? Scientific organisations)?

- Agreements with universities
- Take it serious (accreditation): preferably " land" in established educational institutes
- Accreditation is important for doctiors, but not for patiuents
- cme points are important
- Business model more important than organsatonal form (who will pay)
- CME points + accreditation/ Center of Excellence
- First: Connect to overarching initiatives (ACT- EU PA 10)
- master program in RD Researcher (accreditated by a university)
- PhD Program accrediated by a university
- Research training (like Marie Curie) in a university
- via ERNs
- Hosted by help me? research infrastructure es
- They should be recognized by a large number of institutions. CME is important in my opinion
- Incentives credits
- First: Connect to overarching initiatives (ACT- EU PA 10)
- agreements with universities to involve students, agreements with scientific society to provide CME
- Are these courses open to industry colleagues?
- Multi- dimensional approach: Eurordis -> POs EFPIA -> industry Universities -> future professionals Social media - for everyone
- We should have a "ready to use" moidule for EUPATI / summer school and other patient grouyps and use the existing trainin,g "schools" to disseminate to patients
- Need several types of sustainability
- 1. Money / payment
- 2.Institutional
- sustainability: materials should be stored in a repository (not dependent on individual researchers / teachers)
- 3. Personal: how does it Hosted by help me?
- The risk is that the details that biostatisticians find interesting are not relevant to others
- EXpert level - maybe create an executive master specialised on advanced methods, to be followed largely by Virtual sessions but with university denomination
- joint online seminar (with credits at each university)
- Courses offered on an ongoing basis (in one platform), with ECTS points/certificates
- sustainability also requires resources to update information with the latest research
- the need for credit and incentives depends on the length of the training. Regular short just in time training may be a
- make it part of obligatory programmes for specialisations ! efforts national level and European harmonization
- CME is a cost and not a sustainability factor!

Session 2 – Randomisation in RD CTs

Past: What are practical problems with implementation of randomization in RD-trials?

- randomisation has to be hidden to prevent the ethical issue of doctors deciding what to do for their patient

- Does randomisation prevent the sharing of a same placebo leg between various trials
- to many patients are on placebo, feeling that they are "wasted"
- handling of hidden permutations randomisation lists
- the bias is unclear - only in open lists
- imbalance in recruitments in multicentre trials with centers recruiting only very few patients
- Ethical issues related to being allocated to the control arm
- Limited number of subjects in a CT. Lack of knowledge of randomization methods
- knowledge of MDs about randomization
- in rare disease with highly heterogeneous phenotype you may not be certain that baseline characteristics are not different between arms
- Trial designs are often more complex than textbook examples. Also large differences between recruitment yield between centers in a multicenter trial, often not known a priori
- Randomization req. External agency such as pharmacist
- patients not willing to join the CT to avoid being on PCB

Present: Is the current state of knowledge about implementation of randomization sufficient in RD community?

- Probably not. The use of very short acronyms for each type of randomization make it hard to communicate/understand
- Train also Patient representatives
- difficulty may be faced when there is no data for an accurate power calculation
- Many (Most?) people are unaware of the randomisation bias possibility... But making more people aware just increases the risk (and the perception risk)
- No
- Making clear how much of a problem it is not finished yet
- It is not enough.
- randomisation is quite accepted as gold standrd , but the question is not randomisation per se but what the randomised arms are meant to deliver , ie.e what type of treatments they offer and if there is a placebo control or not !
- With the use of CTs with innovative tools/methods, such as remote elements, there is the need to take this account through training/education
- There is a lot of complex statistical work on pubmed and other scientific platforms, but which method to select is equally important., besides access to stateoftheart research on randomization

Future: What are practical problems with implementation of randomization in RD-trials?

- Deal with temporal Correlation structure in n-of-1 trials (crossover design).
- Prevent n=0- groups.
- Lack of knowledge of randomization techniques and lack of subjects
- in paediatrics usually randomisation against placebo if associated with unpleasant drug delivery is not welcomed
- Need to inform/educate the investigators and also to know where patients could be recruited
- depnds on the disease indicaton and if there is alraedy a standard of care to be used as a comparator
- Combining with other aspect of optimal experimental design.
- Will randomization depend on the analysis method (I guess so).

- before you can randomise, you need to have enough patients participating...
- ultra- rare cases

Session 3 – Endpoints in RD CTs

What issues challenge you in using multiple endpoints in your research?

- sample size infaltion when corecting for multiplicty vs. lack of interpretability for composite endpoint
- some endpoints may not be measured in all participants due to age or mental abilities
- ethical committee & stat experts not aquainted - not a generally accepted standard methodology
- Some endpoints are not chosen correctly and therefore make CTs flounder by being unable to capture patient benefit
- how to design the endpoint structure that will maximise the ability to discriminate working vs. Not working drugs with the lowest possible number of patients
- heterogeneous phenotypes in rare disease
- heterogeneous terminology
- Heterogenity of endpoint types (clinical scales, patient surveys, fluid biomarker levels, imaging data, ...) with some of them assessing different aspects of the disease/treatment effect
- The development of multiple endpoint models is more challenging than just one endpoint with many more considerations needed
- Evaluate the weight of each end point
- The individual contribution of items is hidden in the composite score.
- There is a huge diversity of endpoints being used in some RDs; harmonization would be highly desiirabe
- Defining the components of the multiple endpoint
- To assign the importance to every endpoint. Validation of multiple endpoints. Time to perform different scales
- If we can have quantitative and qualitative end points sums
- some endpoints may not be measured in all

What innovations regarding Multiple Endpoints are necessary for RD clinical trials?

- Define which of those endpoints can be evaluated at home (remotely).
- tools to validate / simulate alternative grouping of multiple effects BEFORE starting the CT
- Introduce more PCOMs and PRO in the multicomponent
- better understanding of how to build and use multiple endpoint models ... especially in longitudinal modeling.
- Some endpoints are not chosen correctly and therefore make CTs flounder by being unable to capture patient benefit
- analysis in finite population clinical trials with randomized inference
- Engage up front more with Reg authorities on the use of relevant endpoints in RD
- Educate clinicians on this possibility as in many diseases it is more than1 end point
- tools to better understnad placebo effects
- illustrate methods by examples
- link to other components of trial design like randomization

- how can we incorporate relevant endpoint, such as development or cognition in trials in RD considering the usual duration of a trial and possible time needed for change in outcome/ endpoint
- Validation procedures suited for small sample size
- better definitions of clinical relevance for patients
- Training
- guidance concept paper approved by regulators and ethical committees

Which types of Multiple Endpoints are most helpful in the context of your RD clinical trials?

- Item response models (estimating underlying hidden variable of disease status based on multiple measurements of that disease).
- Unclear - we need tools to understand how to select the best ones
- Goal attainment scales
- Usually well chosen quantitative, objective measures of patient benefit are helpful, but also QoL surveys can tell a lot
- Sensitive measures capturing actual clinical benefit rather than a proxy measure.
- Composite endpoints
- Endpoints usually need to evaluate both motor and cognitive components. Endpoints should always be individualized for every clinical trial.
- multiple endponts embracing EFS and biological parameters and QoL scores
